# Catabolic Effects of Human PTH (1–34) on Bone: Requirement of Monocyte Chemoattractant Protein-1 in Murine Model of Hyperparathyroidism

**DOI:** 10.1038/s41598-017-15563-7

**Published:** 2017-11-10

**Authors:** Jawed A. Siddiqui, Joshua Johnson, Carole Le Henaff, Claudine L. Bitel, Joseph A. Tamasi, Nicola C. Partridge

**Affiliations:** 1 Department of Basic Science and Craniofacial Biology, New York University College of Dentistry, New York, New York USA; 2 Bristol-Myers Squibb, Pennington, New Jersey USA

## Abstract

The bone catabolic actions of parathyroid hormone (PTH) are seen in patients with hyperparathyroidism, or with infusion of PTH in rodents. We have previously shown that the chemokine, monocyte chemoattractant protein-1 (MCP-1), is a mediator of PTH’s anabolic effects on bone. To determine its role in PTH’s catabolic effects, we continuously infused female wild-type (WT) and MCP-1^−/−^ mice with hPTH or vehicle. Microcomputed tomography (µCT) analysis of cortical bone showed that hPTH-infusion induced significant bone loss in WT mice. Further, μCT analysis of trabecular bone revealed that, compared with the vehicle-treated group, the PTH-treated WT mice had reduced trabecular thickness and trabecular number. Notably, MCP-1^−/−^ mice were protected against PTH-induced cortical and trabecular bone loss as well as from increases in serum CTX (C-terminal crosslinking telopeptide of type I collagen) and TRACP-5b (tartrate-resistant acid phosphatase 5b). *In vitro*, bone marrow macrophages (BMMs) from MCP-1^−/−^ and WT mice were cultured with M-CSF, RANKL and/or MCP-1. BMMs from MCP-1^−/−^ mice showed decreased multinucleated osteoclast formation compared with WT mice. Taken together, our work demonstrates that MCP-1 has a role in PTH’s catabolic effects on bone including monocyte and macrophage recruitment, osteoclast formation, bone resorption, and cortical and trabecular bone loss.

## Introduction

PTH has multifaceted effects on bone, depending on the mode of administration. *In vivo*, intermittent administration of PTH increases bone mass by increasing the number and activity of osteoblasts, while a continuous infusion of PTH decreases bone mass by causing a net increase in bone resorption. Overproduction of PTH produces the pathophysiological disorder, hyperparathyroidism (HPT) which is characterized by hypercalcemia, hypercalciuria and increases in osteoclastic bone resorption^1^. PTH regulates osteoblast and osteoclast activity through various mechanisms including the up-regulation of receptor activator of nuclear factor kappa-B ligand (RANKL) and down-regulation of osteoprotegerin (OPG) expression by osteoblasts. However, the early molecular targets or factors that initiate and/or mediate the catabolic response to PTH are not well known due to limited *in vivo* data.

It has been well established that soluble mediators of immune cell function including chemokines, cytokines, and several growth factors are required for the regulation of osteoblast and osteoclast activity and are also necessary for the PTH-mediated effect on bone. Monocyte chemoattractant protein-1 (MCP-1 or CCL2), originally termed JE, is a CC chemokine, and plays a crucial role in the recruitment and activation of leukocytes during inflammation^2^. MCP-1 is highly expressed at sites of osteoporotic bone^3^ and in prostate cancer-induced bone resorption^4,5^. Further, PTH related-peptide (PTHrP) was shown to stimulate MCP-1 production by human bone marrow endothelial cells and osteoblasts resulting in increased osteoclast differentiation, prostate cancer cell proliferation and invasion *in vitro* ^6^. MCP-1 is also expressed by osteoclasts and stimulates osteoclast formation^7–9^. MCP-1 binds to CC chemokine receptor 2 (CCR2), which is expressed by osteoclasts^8^, and absence of CCR2 results in higher bone mass^10^, strongly suggesting a role for MCP-1 in bone metabolism.

Both intermittent and continuous administration of PTH differentially regulates many genes^11,12^. In our laboratory, microarray studies of bone mRNA from rats treated with intermittent hPTH(1–34) showed that the chemokine, MCP-1, was the most highly induced gene (100–250 fold) and was produced by osteoblasts in response to PTH^11,12^. Through further *in vivo* studies, we have shown that rats injected with hPTH(1–34) showed a robust transient increase in serum MCP-1 levels 2 hours after PTH injection compared with basal levels^13^. Furthermore, PTH also increased the number of bone marrow macrophages. As well, MCP-1^−/−^ mice injected daily with hPTH(1–34) did not show an increase in trabecular bone volume and bone mineral density compared with WT mice, concluding that osteoblast MCP-1 expression is an essential mediator of the anabolic effects of PTH on bone^13^.

In the present study, we focused on the role of MCP-1 in PTH’s catabolic effects on bone. We assessed the role of MCP-1 in PTH-induced osteoclast formation by comparing the ability of PTH to increase osteoclasts from *in vitro* bone marrow cultures and recruitment of osteoclasts *in vivo* from WT and MCP-1^−/−^ mice after 2 weeks of hPTH infusion. We show that the ability of PTH to increase osteoclast formation *in vitro* is markedly impaired in cells from MCP-1^−/−^ mice. We found that MCP-1 is necessary for osteoclast and macrophage recruitment, osteoclast formation and bone resorption, pathological consequences associated with PTH’s catabolic effects. We, therefore, conclude that MCP-1 is an important chemokine in PTH-induced osteoclast formation and bone resorption. Our studies provide evidence that in hyperparathyroidism, continuous up-regulation of MCP-1 expression is required for PTH-mediated catabolic effects on bone.

## Results

### MCP-1 null mice are protected from continuous PTH-induced cortical bone loss in a mouse model of primary hyperparathyroidism

**Figure 1:**
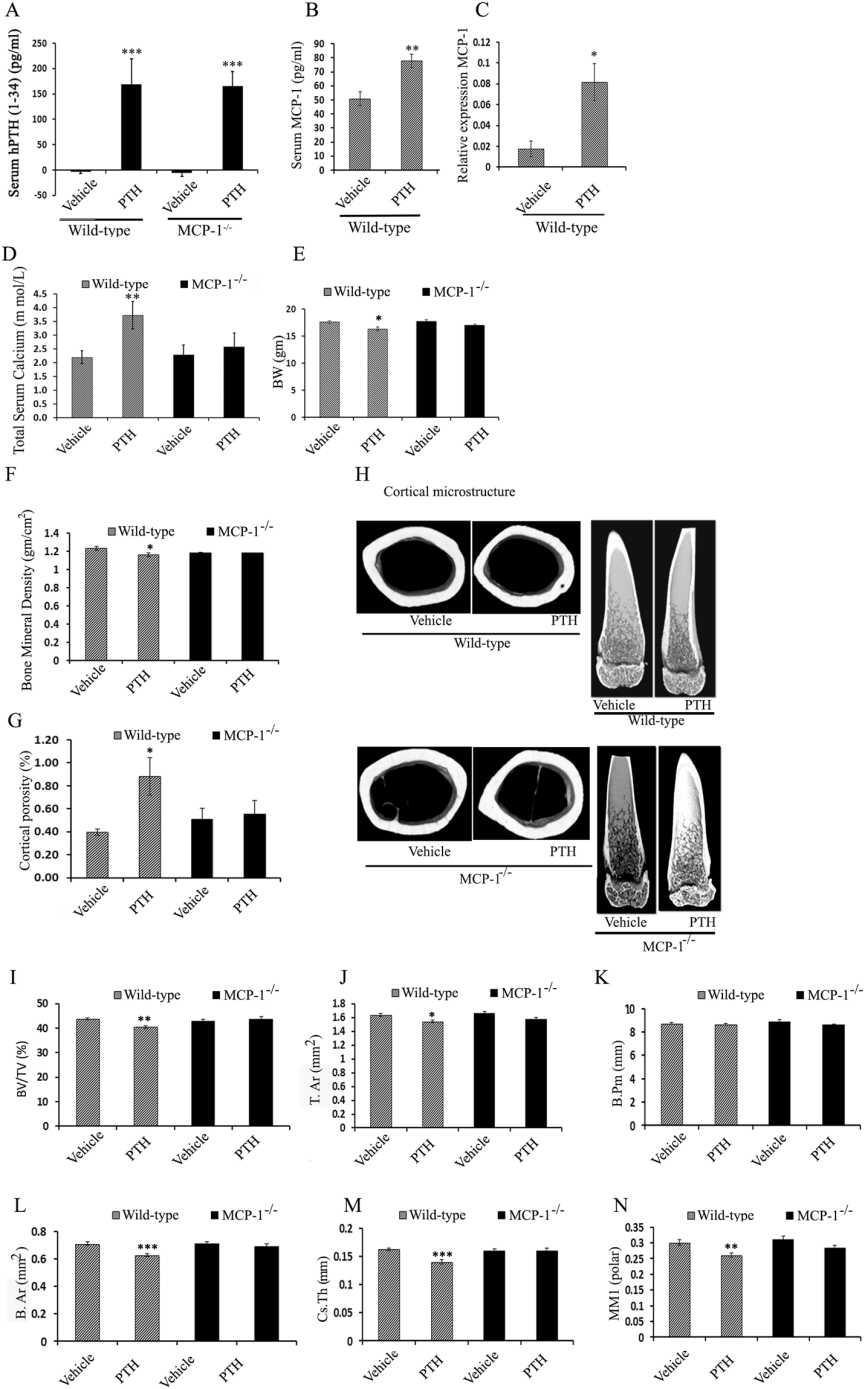
MCP-1 is required for cPTH-induced cortical bone loss. (**A**) Effect of continuous hPTH infusion on serum hPTH levels. Serum hPTH significantly increased in WT and MCP-1^−/−^ mice. (**B**) Serum MCP-1 levels are significantly increased in WT mice after cPTH infusion. (**C**) MCP-1 gene expression after cPTH infusion. (**D**) Total serum calcium (mmol/L). (**E**) Change in body weight (BW) in gram (gm). (**F**) Cortical bone mineral density (vBMD cortical) in (gm/cm^3^). MCP-1^−/−^ mice did not show loss of cortical BMD after cPTH infusion as seen in WT mice. (**G**) cPTH infusion increased cortical porosity in WT mice while not affecting this parameter in MCP-1^−/−^ mice. (**H**) Representative µCT images of the cortical region of the femurs. (**I–N**) µCT analysis of cortical bone in the femurs of mice showing BV/TV (%), T.Ar (mm^2^), B.Pm (mm), B.Ar (mm^2^), Cs.Th (mm) and MMI polar. All values are expressed as mean ± SEM (n = 10–14 mice/group) *p < 0.05, **p < 0.01 and ***p < 0.001 compared to vehicle-infused WT mice.

To ascertain the constant elevation of PTH levels, as seen in primary hyperparathyroidism (HPT), we infused hPTH (1–34) to WT and MCP-1^−/−^ mice for 2 weeks, referred to hereafter as continuous PTH (cPTH). To establish and validate the catabolic effect of cPTH, we measured serum levels of hPTH (1–34). As expected, no hPTH (1–34) was detected in either WT or MCP-1^−/−^ mice infused with saline, whereas, after 2 weeks, PTH-infused mice exhibited significant circulating levels of hPTH (1–34) (Fig. 1A). Similarly, we did not find serum MCP-1 in vehicle or cPTH- infused MCP-1^−/−^ mice (data not shown). There was a 1.5 to 2-fold increase in serum MCP-1 levels in PTH-infused WT mice compared to vehicle-infused WT mice (Fig. 1B). Further, 2 weeks of cPTH infusion up-regulated MCP-1 expression (4 fold) in the distal femurs of WT mice (Fig. 1C). Continuous infusion of PTH significantly increased serum calcium concentrations in WT mice but not in MCP-1^−/−^ mice (Fig. 1D).

In WT mice, cPTH decreased body weight (BW), while it did not in MCP-1^−/−^ mice (Fig. 1E). MicroCT analysis of cortical bone (midshaft femur) showed that cPTH caused significant decreases in cortical vBMD in WT animals. However, MCP-1 null mice did not show a reduction in vBMD after cPTH infusion, similar to our observation of the role of MCP-1 in PTH’s anabolic effects^13^ (Fig. 1F). Cortical porosity increased in WT mice after cPTH infusion while MCP-1 null mice did not show this increase (Fig. 1G). In WT mice, cPTH infusion induced significant decreases in cortical bone volume (BV/TV), total area (T.Ar), bone area (B.Ar), total cortical thickness (Cs.Th) and polar mean moment of inertia (MMI). However, we did not observe any of these changes in MCP-1^−/−^ mice, suggesting that MCP-1 is a major factor in the cPTH-induced cortical thinning and loss of cortical volume in wild-type animals (Fig. 1I–N). In addition, the WT mice showed a significant increase in the endocortical perimeter with cPTH, indicating an increase in resorption in this area (Supplementary Data, Fig. 1).

### Deletion of MCP-1 blunts PTH-mediated trabecular bone loss

**Figure 2:**
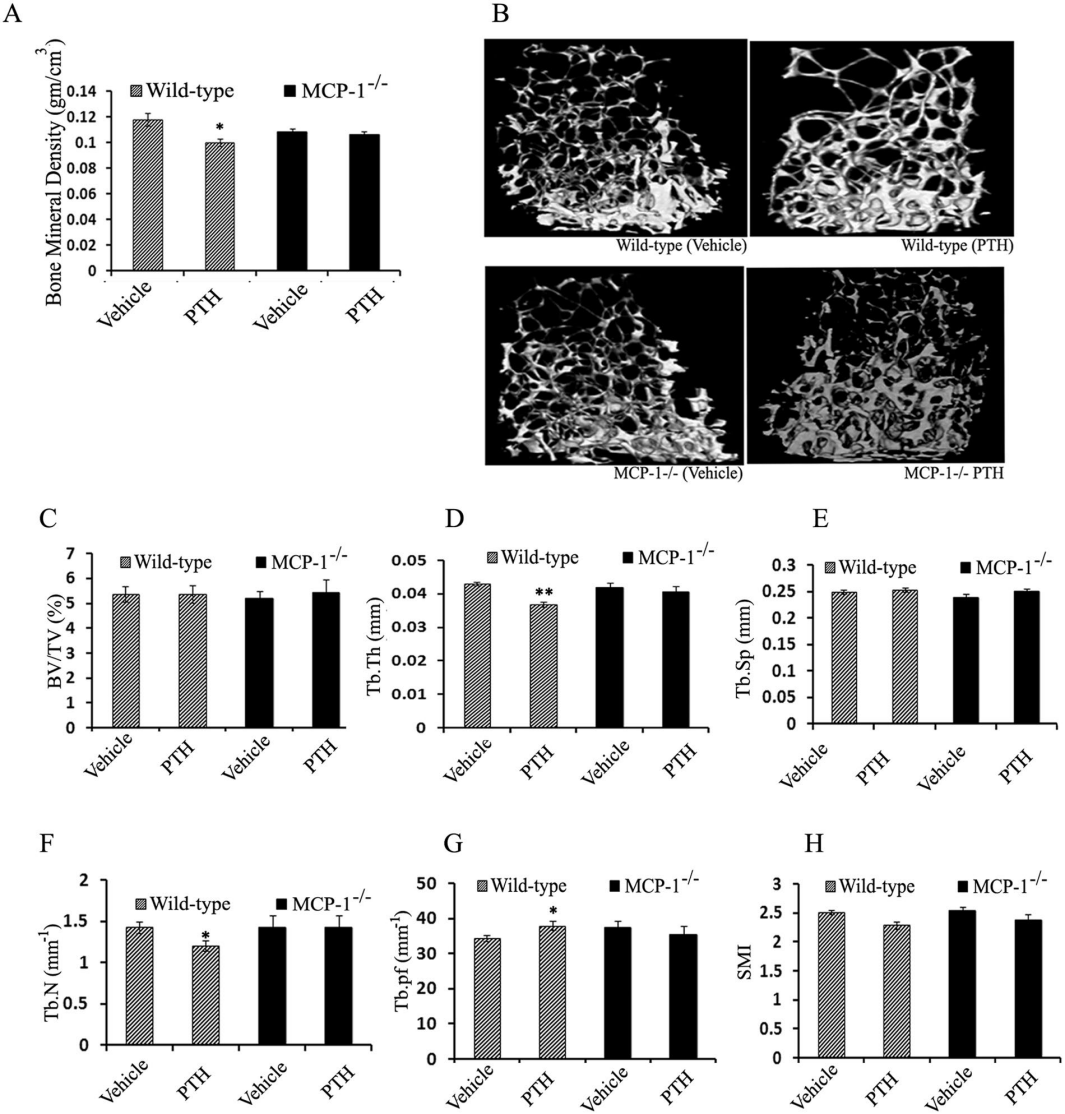
MCP-1 null mice are protected from cPTH-induced trabecular bone loss. (**A**) Trabecular bone mineral density (vBMD trabecular) in (gm/cm^3^). (**B**) Representative µCT images of the trabecular region of femurs. (**C–H**) µCT analysis of trabecular bone in the femurs showing BV/TV (%), Tb.Th (mm), Tb.Sp (mm), Tb.N (1/mm), Tb.Pf (1/mm) and SMI. All values are expressed as mean ± SEM (n = 10–14 mice/group). *p < 0.05 and **p < 0.01 compared to vehicle-infused WT mice.

MicroCT analyses of trabecular bone showed a decrease in vBMD of the femur trabecular region in WT mice after continuous PTH infusion, while MCP-1^−/−^ mice did not show changes (Fig. 2A). Three-dimensional µCT analysis indicated that the PTH infusion led to sparsity in the architecture of the secondary spongiosae of trabecular bone of WT mice. In contrast, the MCP-1 deficient mice did not demonstrate any pattern changes after cPTH infusion (Fig. 2B).

At baseline, there were no significant differences in µCT parameters between MCP-1 null and WT mice. Femoral trabecular bone volume (BV/TV) and trabecular separation (Tb.Sp) were similar in cPTH and vehicle-infused WT and MCP-1^−/−^ mice (Fig. 2C and E). However,

MCP-1^−/−^ mice did not show the decrease in femoral trabecular thickness (Tb.Th) and trabecular number (Tb.N) with cPTH infusion which were seen in WT mice (Fig. 2D and F). Trabecular pattern formation (Tb.Pf) is a geometric parameter, and higher values indicate disconnected trabecular structure. As expected, Tb.Pf is greater in cPTH-infused WT mice. There were no changes in Tb.Pf in cPTH-infused MCP-1^−/−^ mice. These results showed that deletion of MCP-1 protected the animals from the cPTH-mediated trabecular deterioration (Fig. 2G).

### No recruitment of osteoclasts in MCP-1^−/−^ mice treated with PTH

**Figure 3:**
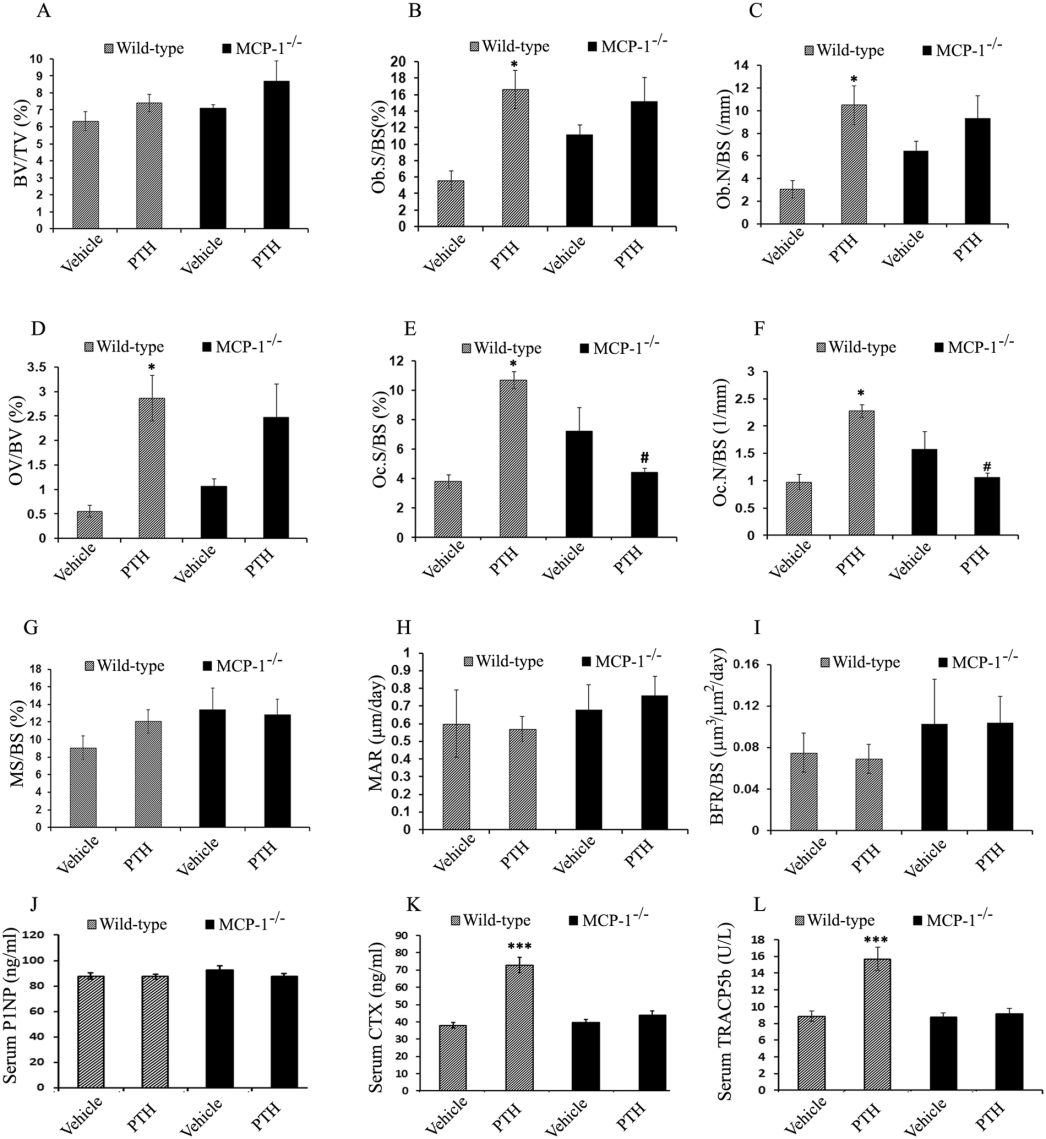
Histomorphometric analysis of femurs of mice (WT and MCP-1^−/−^) infused with cPTH for 2 weeks and biochemical markers. (**A–F**) Static histomorphometric analysis of femurs of WT and MCP-1^−/−^ mice revealed a significant increase in osteoblast surface (Ob.S), osteoblast number (Ob.N), osteoid surface (OV/BV), osteoclast surface (Oc.S) and osteoclast number (Oc.N) in WT mice after 2 weeks of cPTH infusion. In contrast, cPTH-stimulated osteoblast and osteoclast activities are abolished in MCP-1 null mice. (**G–I**) There are no significant changes in analyzed dynamic histomorphometric parameters. (n = 6 mice/group). (**J**) The levels of P1NP, a marker of bone formation is unchanged in all groups; (**K**) CTX, a marker of bone resorption, (**L**) TRACP5b (surrogate for osteoclast number), a marker of bone resorption are increased with cPTH in WT mice but not in MCP-1^−/−^ mice. All values are expressed as mean ± SEM (n = 10–14 mice/group). *p < 0.05 and ***p < 0.001 compared to vehicle-infused WT mice. ^#^p < 0.05 compared to PTH-infused WT mice.

Histomorphometric indices of osteoblasts and osteoid production were significantly augmented with cPTH infusion in WT mice as has been well-documented by us and others^11,14^. The ratio of bone surface covered by osteoblasts (Ob.S/BS), numbers of osteoblasts (Ob.N/BS), and osteoid volume (OV/BV) were all significantly elevated in cPTH-infused WT mice, but not in MCP-1 deficient mice (Fig. 3B–D), similar to our observations with intermittent injections of PTH. In contrast, the osteoclast surface per bone surface (Oc.S/BS) and number of osteoclasts/bone surface (Oc.N/BS), indices of bone resorption, were highly increased (2.5-fold) in the cPTH-infused WT mice, showing cPTH-induced trabecular bone resorption. These increases in bone resorption indices were completely absent in the MCP-1 null mice showing an absence of recruitment of osteoclasts (Fig. 3E,F). Dynamic parameters demonstrate that mineralizing surface (MS/BS), mineral apposition rate (MAR) and bone formation rate (BFR/BS) do not differ between wild-type and MCP-1^−/−^ mice in both vehicle and cPTH-infused groups. (Fig. 3G–I).

As shown in Fig. 3J, serum P1NP levels were similar in both WT and MCP-1^−/−^ mice even after cPTH infusion. Serum CTX levels increased in WT mice after cPTH infusion. However, there was no change in serum CTX levels in MCP-1^−/−^ mice (Fig. 3K). Similarly, the levels of serum TRACP-5b in WT mice increased significantly in the PTH-infused groups compared with the vehicle-infused groups (Fig. 3L). However, there was no stimulation of serum TRACP-5b concentrations in PTH-infused MCP-1^−/−^ mice. Both these latter parameters paralleled the lack of recruitment of osteoclasts after cPTH in the MCP-1^−/−^ mice.

### MCP-1 is required for PTH-induced osteoclast formation and bone resorption

**Figure 4:**
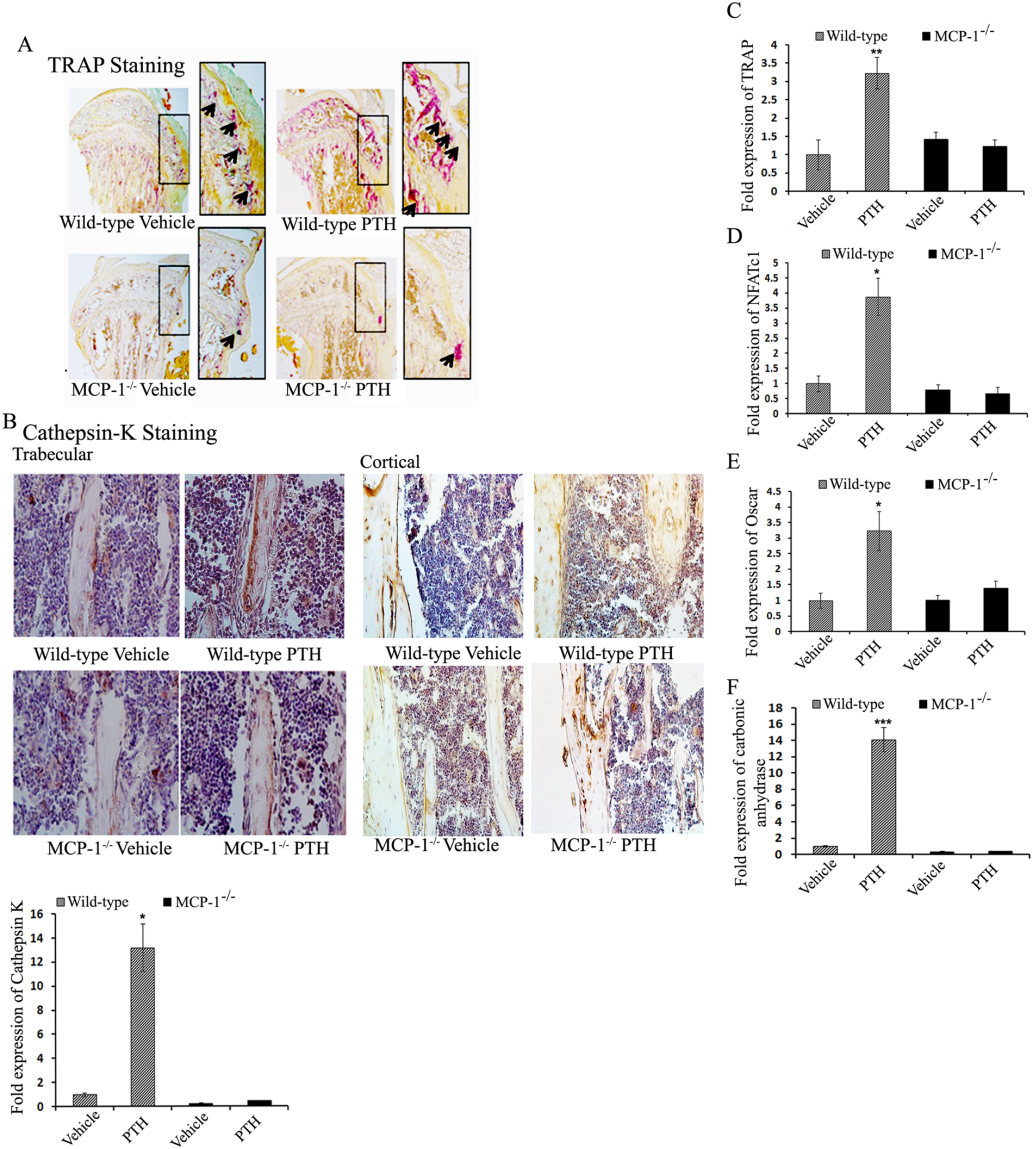
MCP-1 is necessary for cPTH-mediated osteoclastogenesis and osteoclast activity. (**A**) TRAP staining of tibiae from WT and MCP-1^−/−^ mice (magnification 20X) shows less osteoclasts in MCP-1^−/−^ mice with or without cPTH. (**B**) Immunohistochemistry for cathepsin K (known to be a more specific marker of osteoclasts) confirms the observations in (**A**). Lower panel, cathepsin K mRNA expression after PTH infusion. (**C–F**) mRNAs extracted from distal femur metaphyses and RT-qPCR was performed to examine the expression of TRAP, NFATc1, Oscar and carbonic anhydrase. No increase in any of these genes was seen in the MCP-1^−/−^ mice infused with PTH in contrast to the WT mice. *p < 0.05, **p < 0.01, ***p < 0.001 compared with vehicle-infused WT mice.

Continuous PTH significantly increased the number of tartrate-resistant acid phosphatase (TRAP)-positive cells in WT tibiae; however, no such cPTH-induced increase in TRAP-positive cells was observed in MCP-1-deficient tibiae (Fig. 4A). Further, immunohistochemistry for cathepsin K, a marker of mature osteoclasts, revealed that MCP-1 null mice did not show the increase in cathepsin K-positive cells after cPTH infusion seen in WT mice (Fig. 4B).

In line with these analyses, we performed qPCR analysis for osteoclast genes in the distal femur. Continuous PTH failed to induce expression of cathepsin K mRNA in bones of MCP-1^−/−^ mice while there was a 12-fold increase seen in the WT mice (Fig. 4G). Similarly, deletion of MCP-1 abolished the cPTH-induced up-regulation of other osteoclast-specific genes (TRAP, NFATc1, Oscar and carbonic anhydrase) (Fig. 4C–F). These results show that the absence of MCP-1 prevented PTH-induced increases in bone resorption by abolishing the increase in the number of osteoclasts in the local milieu of bone.

### MCP-1 is required for PTH-mediated increases in macrophages and monocytes

**Figure 5:**
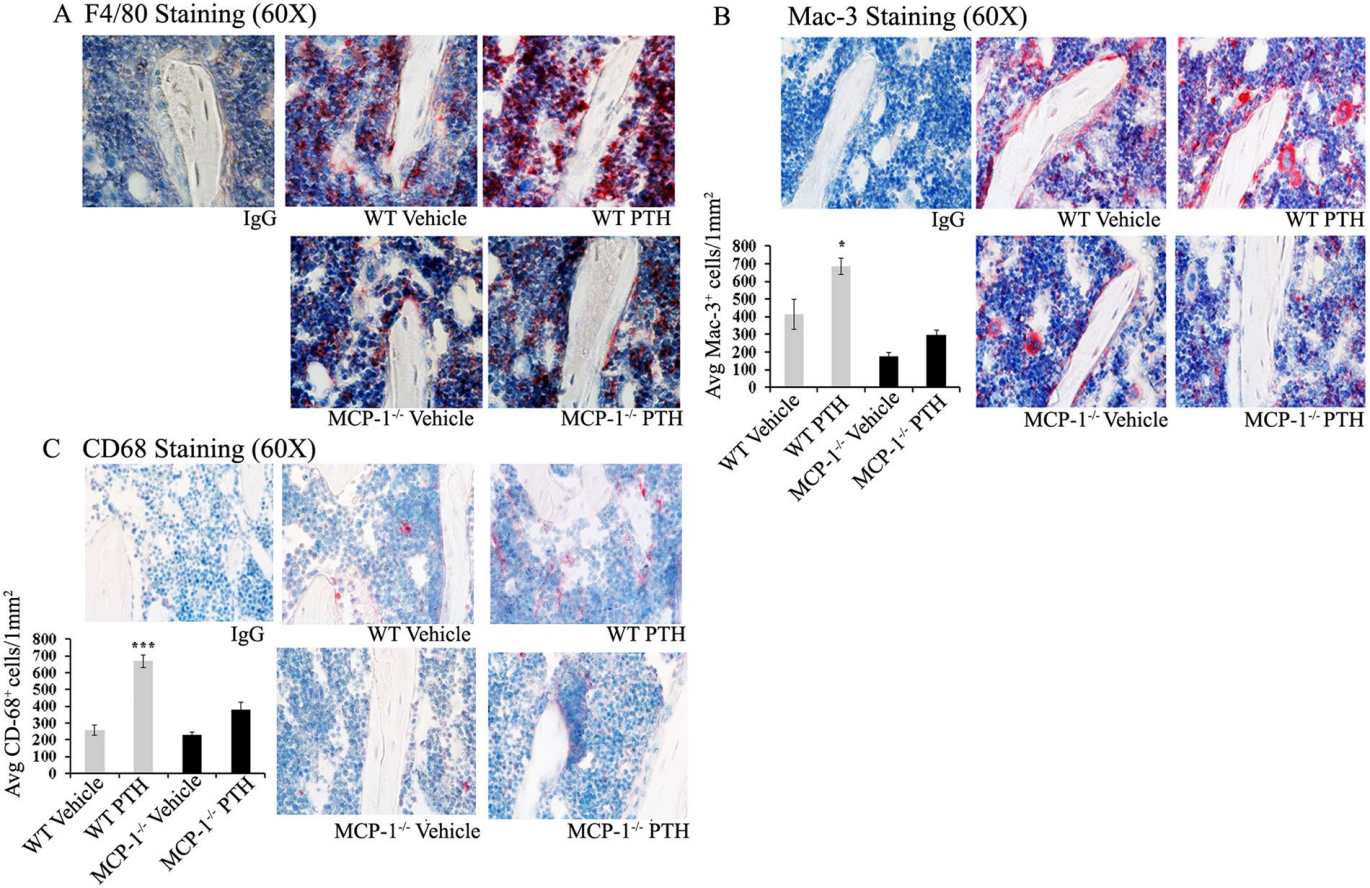
Continuous PTH recruits increased numbers of macrophages in bone marrow and this is ablated in MCP-1^−/−^ mice. (**A**) Two weeks of cPTH infusion increased the osteomac population in WT mice compared to vehicle-infused WT mice. MCP-1^−/−^ mice showed fewer F4/80 positive cells in both vehicle and PTH-infused groups. Immunohistochemistry of tibial sections with F4/80 (magnification 60X). (**B**) Sections of WT and MCP-1^−/−^ mice immunostained for Mac-3 and quantitation of stained cells. **(C)** CD68 immunostained sections of tibiae of WT and MCP-1^−/−^ mice infused with cPTH or vehicle and quantitation of stained cells. There is a modest expression of Mac-3 and CD68 in MCP-1 null mice, with and without cPTH infusion (magnification 60X) and no stimulation with PTH. IgG was used as negative control for each stained section. *p < 0.05, ***p < 0.001 compared to vehicle-infused WT mice.

As we have previously observed, PTH also stimulates numbers of bone marrow macrophages^13^. Immunohistochemical analyses of F4/80 expression demonstrated greater staining for F4/80-positive macrophages in cPTH-infused tibial sections of WT mice. MCP-1^−/−^ mice showed fewer F4/80 positive cells in both vehicle and PTH-infused groups (Fig. 5A). By using the mouse macrophage-specific marker, Mac-3, we found significant increases in numbers of cells stained for this marker in the secondary spongiosae after cPTH infusion in WT mice but low staining in MCP-1^−/−^ mice even after cPTH infusion, demonstrating that MCP-1 is necessary for monocyte and macrophage recruitment in bone marrow (Fig. 5B). Similarly, MCP-1^−/−^ mice showed lesser CD68 positive cells in either vehicle or cPTH-infused mice (Fig. 5C).

### cPTH-mediated *in vivo* osteoclastogenesis is independent of the RANKL/OPG axis

**Figure 6:**
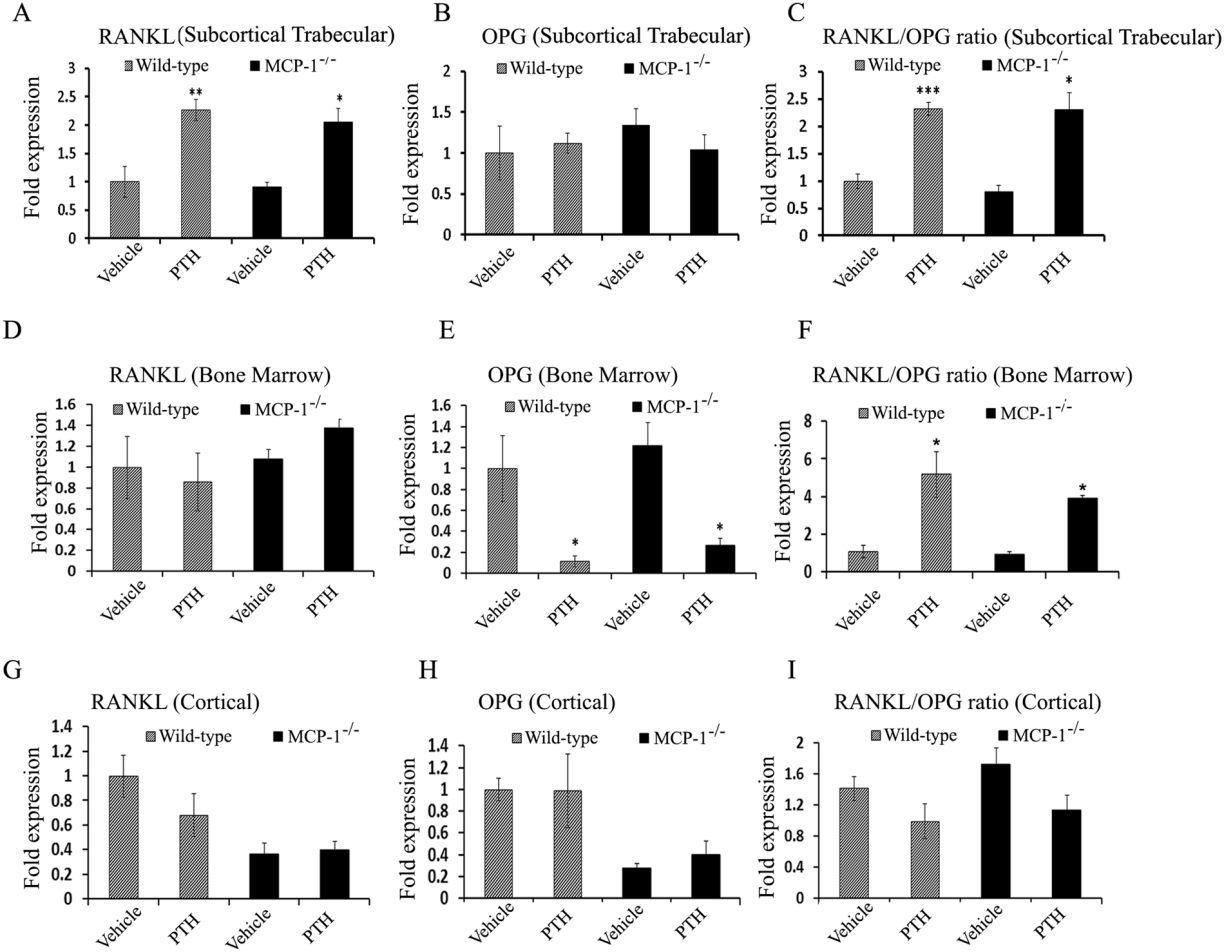
MCP-1 regulates cPTH-induced bone loss independent of the RANKL/OPG axis. WT and MCP-1^−/−^ mice were infused with cPTH for 2 weeks. Subcortical trabecular, bone marrow and cortical bone compartments were separated, and RT-qPCR was performed to determine the differential expression of RANKL and OPG in these different compartments. (**A,B,C**) Increased expression of RANKL in subcortical trabecular bone after cPTH in both WT and MCP-1^−/−^ mice, (**A**) RANKL, (**B**), OPG, (**C**), RANKL/OPG ratio. (**D,E,F**) Decreased expression of OPG in bone marrow after cPTH in both WT and MCP-1^−/−^ mice, (**D**) RANKL, (**E**), OPG, (**F**), RANKL/OPG ratio. (**G,H,I**) Expression in cortical bone, (**G**) RANKL, (**H**), OPG, (**I**), RANKL/OPG ratio. (n = 4 mice in each group) *p < 0.05, **p < 0.01, ***p < 0.001 compared to vehicle-infused WT or MCP-1^−/−^ mice.

To investigate whether the requirement of MCP-1 in the catabolic effect of PTH involves the RANKL/OPG axis *in vivo*, we evaluated the mRNA expression of RANKL and OPG, which are known to influence osteoclast formation and function, in different bone compartments (subcortical trabecular, bone marrow and cortical). In the trabecular-rich compartment, we found increased RANKL expression in both WT and MCP-1^−/−^ mice after cPTH infusion while OPG mRNA expression was not altered (Fig. 6A and B). The ratio of RANKL/OPG, which reflects increased resorption and decreased bone mass, significantly increased in both WT and MCP-1^−/−^ mice (Fig. 6C). On the other hand, in the bone marrow compartment, there were no changes in RANKL expression while mRNA expression of OPG decreased after cPTH infusion in both WT and MCP-1^−/−^ mice, thus again the RANKL/OPG ratio significantly increased in this compartment in both WT and MCP-1^−/−^ mice after cPTH infusion, similar to the subcortical trabecular compartment (Fig. 6D,E and F). In cortical-rich bone, cPTH did not significantly change the mRNA expression of either RANKL or OPG in both WT and MCP-1^−/−^ mice compared to vehicle-infused mice (Fig. 6G and H). The RANKL/OPG ratio in cortical bone was also not altered in WT and MCP-1^−/−^ mice after PTH infusion (Fig. 6I). The data suggest that MCP-1 is required for PTH-mediated osteoclast formation and bone resorption by a mechanism independent of the RANKL/OPG system.

### MCP-1 modulates osteoclast differentiation and MCP-1 null mice exhibit impaired PTH- mediated bone marrow osteoclast formation

**Figure 7:**
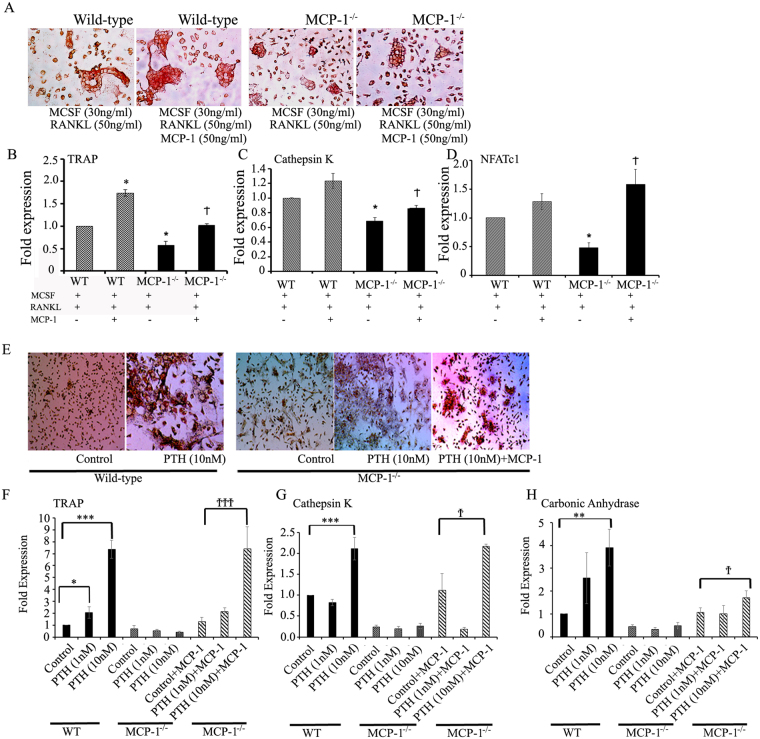
MCP-1 promotes osteoclastogenesis *in vitro* and MCP-1 null mice exhibited impaired PTH-mediated bone marrow osteoclast formation. (**A**) Bone marrow cells from WT or MCP-1^−/−^ mice were induced to differentiate towards the osteoclast lineage with M-CSF and RANKL for 7 days in the presence or absence of exogenous MCP-1 (50 ng/ml). Cultures were stained for TRAP and photographed by light microscopy. (**B**–**D**) RT-qPCR analyses showing mRNA expression of osteoclast markers (TRAP, Cathepsin K and NFATc1) using the same cells as in (A) (n = 3). *p < 0.05 compared to cells from WT mice treated with M-CSF and RANKL, ^†^p < 0.05 compared to MCP-1^−/−^ BMMs treated with M-CSF and RANKL. (**E**) Bone marrows from WT and MCP-1^−/−^ mice were cultured for 10 days in the presence or absence of hPTH(1–34; 10^−9^ or 10^−8^M) and/or MCP-1 (50 ng/ml) to induce osteoclast formation. Cultures were stained for TRAP to identify multinucleated TRAP-positive cells. Representative images by light microscopy (4X). (**F**–**H**) Bone marrows from WT and MCP-1^−/−^ mice were cultured for 10 days in the presence or absence of hPTH(1–34; 10^−9^ or 10^−8^M) and/or MCP-1 (50 ng/ml) to induce osteoclast formation. Isolated RNAs were used for RT-qPCR analysis of osteoclast specific gene expression (TRAP, cathepsin K and carbonic anhydrase). *p < 0.05, **p < 0.01, ***p < 0.001 compared to Control cells from WT mice and ^†^p < 0.05, ^†††^p < 0.001 compared to Control + MCP-1 -treated cells from MCP-1^−/−^ mice.

To further evaluate whether MCP-1 is required for osteoclastogenesis, we generated MCP-1^−/−^ and WT osteoclasts from bone marrow macrophages (BMMs) in the presence of M-CSF, RANKL and/or MCP-1. BMMs from MCP-1^−/−^ mice showed decreased multinucleated osteoclast formation compared to WT mice and exogenous treatment with MCP-1 resulted in an increase in multinucleated osteoclast formation (Fig. 7A). Furthermore, expression of osteoclast-specific genes (TRAP, cathepsin K and NFATc1) was significantly decreased in BMMs from MCP-1^−/−^ mice compared to BMMs obtained from WT mice. Co-treatment with MCP-1 together with M-CSF and RANKL to BMMs from MCP-1^−/−^ mice caused an increase in expression of these genes compared with BMMs from MCP-1^−/−^ mice treated with M-CSF and RANKL (Fig. 7B,C and D).

*In vitro*, PTH treatment (10^−9^–10^−8^ M) significantly increased osteoclast formation from bone marrows of WT mice while failing to increase osteoclast formation in bone marrows derived from MCP-1^−/−^ mice. Representative images of bone marrow cultures stained for TRAP to visualize osteoclasts are shown in Fig. 7E. PTH (10^−8^ M) treatment enhanced the expression of TRAP, cathepsin K and carbonic anhydrase in bone marrow cultures from WT mice but not from cultures from MCP-1^−/−^ mice. Co-treatment with MCP-1 together with PTH (10^−8^ M) to cultures from MCP-1^−/−^ mice significantly augmented the expression of TRAP, cathepsin K and carbonic anhydrase compared to controls receiving MCP-1 (Fig. 7F–H).

## Discussion

Parathyroid hormone is a prototypic osteoanabolic hormone for treating osteoporosis when given as a daily injection. Sustained high serum levels of PTH or continuous infusion of PTH cause a catabolic effect reflected in decreased bone mass due to a net increase in bone resorption^15,16^. In this present study, we investigated whether MCP-1 might participate in the catabolic effects of PTH. We found that ablation of the MCP-1 gene prevents the catabolic effects of cPTH on bone, and that the recruitment of monocytes, macrophages and osteoclasts are impaired in the null mice with cPTH infusion. PTH increases renal reabsorption of calcium and also produces net bone resorption with the resultant mobilization of skeletal calcium and finally an increase in serum calcium levels^17,18^. Our data demonstrated that MCP-1^−/−^ mice did not show an increase in serum calcium levels after PTH infusion unlike that seen in WT mice.

In the present study, we did not find a basal developmental bone phenotype in MCP-1^−/−^ mice. This observation has been reported previously^10,13^. However, MCP-1 is only one of several ligands for CCR2^19^ and redundancy in the chemokine system may contribute to the migration of preosteoclastic cells to allow normal bone development. The need for MCP-1 may only be apparent with a challenge, such as PTH infusion or injection.

Hyperparathyroidism is associated with a lower bone mass at all skeletal sites, but particularly at sites containing predominantly cortical bone^20^. Continuous PTH infusion or HPT induces cortical bone deterioration at least in part by enhancing endosteal and intracortical bone resorption^21,22^. Although severe, persistent elevations of PTH levels may also lead to trabecular bone loss^21^, both HPT and continuous PTH treatment usually exhibit a modest increase in trabecular bone^23,24^. Thus, bone densitometry studies suggest that the response to elevated PTH levels is site specific.

Cortical bone has a significant role in determining bone strength and a loss of cortical bone can comprise either thinning of the cortex or an increase in cortical porosity. Bone remodeling in the cortical region takes place at the endocortical, the pericortical and Haversian surfaces. The pathophysiological discrepancy between intracortical and endocortical remodeling leads to cortical bone porosity. In our studies, continuous infusion of PTH resulted in increased cortical porosity in WT mice and this has been previously reported^22^. Nevertheless, deletion of MCP-1 prevented this effect.

Continuous infusion of PTH is characterized by increased numbers of osteoclasts and rapid bone turnover. In our present results, the significant 2.5-fold increase in osteoclast number and osteoclast surface seen in WT mice infused with PTH was abolished in the MCP-1 null mice, confirming that MCP-1 is a major crucial factor in PTH-induced osteoclast formation. As is well known, we also found increased numbers of osteoblasts and osteoblast surface^11^ as well as increased osteoid volume, but there was no increase in osteoblast activity as indicated by MS/BS, MAR, BFR/BS, suggesting that the mineralization activity per osteoblast was reduced or the period of time was insufficient to observe changes in these parameters. Similarly, patients with HPT are reported to have slightly increased osteoid seam thickness, prolonged osteoid maturation period and decreased trabecular mineralization, with resultant decreased BMC and BMD^25,26^. The mineralization defect may be partly due to the decrease in serum phosphate that these patients exhibit.

MCP-1 null mice have a profoundly reduced ability for recruited macrophages to fuse to form foreign body giant cells^27^, demonstrating a role for MCP-1 in macrophage fusion. F4/80^+^ macrophages, also termed osteomacs, have been reported to reside in the endosteal surface^28^ where they contribute to retaining hematopoietic stem cells in the BM niche and osteoblast function^29^. Continuous PTH infusion did not cause an increase in F4/80-positive macrophages within tibiae of MCP-1^−/−^ mice, whereas the hormone did so in WT mice. The Mac-3 antigen is expressed intracellularly by the bone marrow monocyte lineage and is up regulated during macrophage differentiation. In mice with deletion of MCP-1, there was a failure to show a cPTH-induced increase in Mac-3-positive cells. Our results also show that MCP-1 is required for cPTH-mediated increase in the monocyte/macrophage/osteoclast lineage as detected by CD68 (a member of the lysosome-associated membrane protein family) immunohistochemistry. CD68 expression is critical to the normal morphology and function of osteoclasts and CD68^−/−^ mice have increased trabecular bone and their osteoclasts do not efficiently resorb bone^30^. Taken together, these data indicate that MCP-1 is essential for maintenance of the PTH-induced monocyte/macrophage population in bone and the PTH-mediated increase in osteoclast function.

MCP-1 is produced and acts in an autocrine/paracrine manner to regulate osteoclast formation^7,9^, and osteoclast formation is impaired in mice lacking MCP-1^31^, suggesting that MCP-1 has a major role in the formation and regulation of osteoclasts. In our present study, MCP-1 null mice did not show an increase in TRAP-positive cells in tibial sections, which establishes the role of MCP-1 in PTH-mediated osteoclast recruitment. Cathepsin K-null mice have an osteopetrotic phenotype because of defective bone resorption that is characterized by an increase in the number of faulty osteoclasts and increased bone formation, revealing that cathepsin K is a specific marker of functional osteoclasts^32,33^. Our work showed that MCP-1 is required for the cPTH-mediated increase in cathepsin K expression. In addition to TRAP and cathepsin K, the MCP-1^−/−^ mice did not show an increase in expression of other osteoclast-specific genes in response to cPTH, strongly suggesting that MCP-1 is necessary for cPTH-induced osteoclast formation and function.

It is well established that the catabolic effect of PTH is mediated in part by the enhanced production of the cytokine, RANKL, and by decreased production of the RANKL decoy receptor, OPG, by BMSCs and osteoblasts^16^. It has been suggested that the transient RANKL induction elicited by intermittent PTH administration is associated with its anabolic action, while prolonged induction is related to bone destruction^34^. Here, we have found that cPTH significantly increased the RANKL/OPG ratio (an indicator of bone resorption) in subcortical trabecular bone and bone marrow in both WT and MCP-1^−/−^ mice compared with their vehicle-infused groups. These results demonstrate an important independent role of MCP-1 in cPTH-induced bone destruction and this effect is entirely separate from the RANKL/OPG axis.

*In vitro*, analysis of bone marrow cells (BMMs) from WT and MCP-1^−/−^ mice cultured with M-CSF and RANKL showed significantly less TRAP-positive osteoclasts in MCP-1^−/−^ cultures than WT cultures, and the addition of MCP-1 to MCP-1^−/−^ cultures rescued the TRAP-positive osteoclasts, comparable to WT cultures. Similarly, the mRNA analysis of osteoclast-specific genes showed an analogous pattern. These data strongly suggest that MCP-1 has a role in osteoclastogenesis. *In vitro* PTH treatment did not produce osteoclastogenesis with BMMs derived from MCP-1^−/−^ mice, and the addition of exogenous MCP-1 rescued this effect, further establishing the requirement for MCP-1 in PTH-mediated osteoclastogenesis.

As noted above, the lack of a baseline phenotypic change in the MCP-1^−/−^ mice *in vivo* may be due to redundancy in the chemokine system and because CCR2 has several ligands other than MCP-1^19^. We think that these factors contribute to the migration of preosteoclastic cells to allow normal bone development. The MCP-1^−/−^ mice only show different characteristics when given a stimulus or challenge, such as we have seen with PTH^13^. This has been observed in a number of situations^10,27,35–37^. However, *in vitro* we observe a lack of osteoclastogenesis of BMMs from the MCP-1^−/−^ mice. It should be noted that, *in vitro*, we used RANKL and M-CSF to differentiate the mouse bone marrow cells into osteoclasts. RANKL increases expression of MCP-1 by osteoclasts^7^ and the MCP-1^−/−^ cells *in vitro* would not be able to produce this response. Thus, this may be a contribution to the suppression of osteoclast differentiation *in vitro*.

Besides the differential effects of cPTH infusion in WT and MCP-1^−/−^ mice for many bone parameters as determined by microCT and histomorphometry, the serum marker, P1NP, an indicator of bone formation, did not change between the vehicle and cPTH-infused WT and MCP-1^−/−^ mice. However, deletion of MCP-1 prevented the cPTH-mediated increases in serum CTX and TRACP5b concentrations (reflecting the abundance of osteoclasts, in addition to their activity) seen in WT mice, again showing the requirement of MCP-1 for the cPTH-mediated increase in osteoclast activity and cPTH-induced bone resorption.

Previous studies have demonstrated that MCP-1 plays a pivotal role in stimulating osteoclast formation^7–9,12,38,39^, inducing bone loss in prostate cancer bone metastasis^40–42^, and periodontal bone disease^43,44^. The current studies show the key role of MCP-1 in a model of hyperparathyroidism, which is characterized by accelerated osteoclastogenesis, increased bone turnover and bone loss^45^. In conclusion, our results demonstrate that MCP-1 is required for the effects on bone of hyperparathyroidism due to continuous infusion of PTH. In response to cPTH, osteoblastic MCP-1 recruits and differentiates monocytes, macrophages and osteoclasts. Here we report that mice lacking MCP-1 are protected against the loss of bone micro-architecture, specifically cortical bone, and the stimulation of bone resorption induced by cPTH, thus indicating that MCP-1 is involved in the osteoclastogenic response of PTH. In summary, we have shown here, for the first time that MCP-1 is required for the catabolic bone responses to PTH. This finding has implications for human studies on HPT, and elevated serum MCP-1 has been shown to be correlated with elevated serum PTH levels in women^46^, and to be decreased within minutes after parathyroid adenoma surgery^47^.

## Methods and Materials

### Animals

Breeding pairs of wild-type controls (C57/BL6) and MCP-1^−/−^ mice were purchased from Jackson Laboratories. All animals were kept in a 12-h light/dark cycle with standard rodent chow and water ad libitum. All animal related experimental procedures were performed in accordance with an approved protocol of the Institutional Animal Care and Use Committee of New York University.

### *In vivo* PTH Infusion

Female wild-type and MCP-1^−/−^ mice at 8 weeks of age (n = 10–14 per group) were randomly distributed to vehicle or treatment groups and continuously infused with vehicle (saline) or hPTH (1–34) at 80 μg/kg/day at a delivery rate of 0.25 µl/h for 2 weeks by surgically implanted ALZET osmotic pump model-1002 (DURECT corporation, CA) into the subcutaneous cavity of the mid-scapular region of mice following the manufacturer’s instructions. This age group was selected since it has previously been shown that peak trabecular bone mass is achieved at this age in C57/BL6 mice^48^. Two doses of calcein (10 mg/kg) were given subcutaneously to all groups 2 and 7 days before death.

### Micro-Computed Tomography (μCT)

µCT analysis of excised bones was performed using the Bruker Sky Scan 1172 µCT scanner (Sky Scan, Ltd., Kartuizersweg, Kontich, Belgium). Femurs were dissected from the animals after euthanasia, cleaned of soft tissue, and stored in 70% ethanol. The samples were scanned in batches of six at a nominal resolution (pixels) of 9.7 µm. The following imaging parameters were used: 60 kV, 167 uA, and pixel size of 9.7 µm, matrix size of 2000 × 1000, 0.3 degrees’ rotation, 6 averages, movement correction factor of 10, and aluminum filter. All images were reconstructed using NRECON (Skyscan) with the following parameters: a histogram range of 0-0.065, beam-hardening correction of 40, ring artifact correction of 7, and Gaussian smoothing (factor 1). The reconstructed data were binarised using a thresholding of 79–255. All three-dimensional volumetric analyses of trabecular bone and two-dimensional analyses of cortical bone were performed using the CTAn software (Skyscan). The bone mineral density of trabecular and cortical bone was determined from the binary data based on a calibration curve of calcium hydroxyapatite standards. The μCT measurements follow the guidelines reported by Bouxsein *et al*.^49^. Three-dimensional and two-dimensional analyses were done to determine trabecular and cortical bone architecture respectively. To maintain the consistency of cortical parameters, 100 slices were chosen in the cortical region, omitting 250 slices as offset from the start of the growth plate (to exclude the trabecular region) as a reference point^50^.

### Histomorphometry

Histomorphometry of femurs was performed following our previously described protocol^13^. Briefly, femurs were fixed in 70% ethanol and embedded in methyl methacrylate (Polysciences, Warrington, PA). Longitudinal tissue sections (5 and 10 μm) were cut on a Leica Polycut S microtome. BioQuant image analysis system (Nashville, TN) was used for quantitative histomorphometry. We have scored and quantified fifty fields in the secondary spongiosa at 40X. For static histomorphometry measurement, 5 μm sections were stained with Goldner’s trichrome stain. Fluorochrome (calcein) labeled unstained 10 μm sections were used for dynamic histomorphometry. All histomorphometry measurements were calculated and performed following the standard nomenclature approved by the American Society for Bone and Mineral Research^51^.

### Serum Biochemistry

At the end of the *in vivo* experiments (2 weeks of continuous hPTH infusion), blood samples were collected by cardiac puncture from the mice. Blood was allowed to clot at room temperature and sera collected after centrifugation of blood samples at 5000 rpm for 10 min. Serum MCP-1 concentrations were measured by using the Quantikine ELISA (R&D Systems). Serum C-terminal crosslinking telopeptide of type I collagen (CTX, Immunodiagnostic Systems Inc.), P1NP (MyBiosource.com) and TRACP-5b (Immunodiagnostic Systems Inc.) were measured by rodent-specific ELISAs. Serum total calcium was measured by calcium colorimetric assay kits (BioVision) in accordance with the manufacturer’s instructions.

### Immunohistochemistry

For cathepsin K immunohistochemistry, the paraffin sections (5 μm) of decalcified tibiae were deparaffinized, rehydrated and immunostained with a 1:75 dilution of anti-rat cathepsin K antibody or isotype control (Millipore, Billerica, MA) with the ABC staining system (Santa Cruz Biotechnology) as described previously^13^. Briefly, primary antibodies were incubated overnight at 4 °C followed by 30 minutes’ incubation with secondary antibody at room temperature. For CD68[ED-1] immunohistochemistry, sections were incubated with a 1:50 dilution of anti-rat CD68 [ED-1] (Millipore) for 60 minutes at room temperature followed by 10-minute incubation with secondary antibody with HistoMouse Max AEC detection kit (Invitrogen). AEC chromogen for CD68 detection was applied for 7 minutes and the sections were counterstained with hematoxylin for 1 minute. For F4/80 (Abcam, Cambridge, USA) and Mac-3, the sections were treated similarly to the CD68 sections with a 1:2000 dilution of anti-mouse F4/80 or 1:100 dilution of Mac-3 antibody or isotype control (Serotec, Oxford, UK). CD68 and Mac-3 positive cells were counted in five randomly selected fields from 4 bone sections of each group and Image-J software was used for quantification.

### TRAP Staining

For TRAP staining, cells were fixed with 4% paraformaldehyde in PBS for 30 min at room temperature and stained with the Leukocyte Acid Phosphatase Kit (Sigma) following the company’s instructions.

### Bone Marrow Cell Cultures

Mouse bone marrow cells were isolated as described previously^52^. Briefly, tibiae and femora from 4-week-old WT and MCP-1^−/−^ mice were dissected free of soft tissues. The bone ends were cut, and the marrow cavity was completely flushed with α-minimal essential medium (α-MEM) without serum by using a sterile 25-gauge needle. Marrow cells were cultured in α-MEM containing 10% fetal bovine serum (Hyclone, Logan, UT). Cultures were fed every 2–3 days by replacing 80% of the medium with fresh medium. M-CSF (30ng/ml) and RANKL (50ng/ml) were added at the start of cultures for osteoclasts and at every medium change for 7 days. Where indicated, hPTH(1–34) (10^−8^–10^−9^ M) or control medium were added at the start of bone marrow cultures and at every medium change. MCP-1 (50 ng/ml) or vehicle was added to cells at the start of the cultures and at every medium change. Cells were collected in TRIzol reagent for mRNA analysis. For TRAP staining, cells were fixed on day 7 or 10 of culture with 4% paraformaldehyde. Osteoclast-like cells were defined as TRAP-positive multinucleated cells that contained greater than three nuclei.

### RNA Isolation and Quantitative Real-time PCR Analysis

Femurs from wild type and MCP-1^−/−^ mice were dissected free of soft tissues. In many cases, distal femoral RNA was isolated without removal of marrow. For separation of different bone compartments, bone marrow was completely flushed in an Eppendorf tube with 1.5 mL of phosphate buffered saline (PBS) by inserting a 25-gauge needle into the proximal femur. Once the bone color was changed to completely white from red (without bone marrow), a 2 mm subcortical trabecular rich region was cut from the distal femur. The cortical rich region (2 mm) was cut at mid diaphysis (approximately 5 mm distal to the growth plate). Bone marrow was centrifuged for 2 minutes, and the pellet was stored in RNAlater (Qiagen) at 4 C until RNA isolation. We extracted total RNA from either femurs or cultured cells using a TRIzol kit (Invitrogen). cDNA was synthesized from 1 μg of total RNA using TaqMan® Reverse Transcription Reagents (Life Technologies, Inc.). SYBR® Green Master Mix was used for quantitative real-time RT-PCR using a Mastercycler® ep realplex instrument (Eppendorf). mRNA expression was calculated using a formula reported previously^53^. The levels of mRNA expression were normalized to β-actin expression and then expressed as fold values compared with the WT vehicle-treated mice or the control samples. The details of mouse-specific primers are given in Table 1.

**Table 1:** The mouse-specific primer sequences of various genes used for RT-qPCR.

Gene name	Primer sequence	Accession number
β-actin	F 5′-TCC TCC TGA GCG CAA GTA CTC T-3′ R 5′-CGG ACT CAT CGT ACT CCT GCT T-3′	NM_007393.5
OPG	F 5′-GGC TGA GTG TTT TG GTG GA CAG-3 R 5′-GCT GGA AGG TTT GCT CTT GT GA-3′	NM_008764.3
RANKL	F 5′-TGTACTTTCGAGCGCAGATG-3′ R 5′- ACATCCAACCATGAGCCTTC-3′	NM_011613.3
MCP-1	F 5′-CAT CCA CGT GTT GGC TCA-3′ R 5′- GAT CAT CTT GCT GGT GAA TG AGT-3′	NM_011333.3
OSCAR	F 5′-CAG CTC CAC GGA GAG TGC-3′ R 5′-AGG GGA GGG ACA GCT CAC-3′	NM_175632.3
TRAP	F 5′-GGT CAG CAG CTC CCT AGA AG-3′ R 5′-GGA GTG GGA GCC ATA TGA TTT-3′	NM_001102405.1
Cathepsin K	F 5′-CGA AAA GAG CCT AGC GA ACA-3′ R 5′-TGG GTA GCA GCA GAA AC TTG-3′	NM_007802.4
Nfatc1	F 5′-TCC ACA GTC AT TTG CTC TGC-3′ R 5′-TCC AGC AGG AGG CTA TG TG-3′	NM_016791.4
Carbonic anhydrase	F 5′-TGGTTCACTGGAACACCAAA R 5′-AGCAAGGGTCGAAGTTAGCA	XM_006530050
BSP	F 5′-CCCAGACAGCTGTCCTTCTGAA R 5′-ACGGTGCTGCTTTTTCTGATCG	NM_008318.3
ALPL	F 5′-GTGCCAGAGAAAGAGAGAGA R 5′-TTTCAGGGCATTTTTCAAGGT	NM_001287172.1
SOST	F 5′-AGCCTTCAGGAATGATGCCAC R 5′-TTTGCCGTCATAGGGATGGT	NM_024449.6
F4/80	F 5′-ACAGCTGTACCTGTCAACCAG R 5′-TTGGCCCTCCTCCACTAGAT	X93328.1
Col X	F 5′-TCATGCCTGATGGCTTCATA R 5′-GCACCTACTGCTGGGTAAGC	NM_009925.4

### Statistical Analysis

Statistical analysis was performed by Dunnett’s one-way analysis of variance (ANOVA) or two-way ANOVA followed by Tukey’s multiple comparison test using SigmaStat software (SPSS Sciences, Chicago IL). Data are expressed as the mean ± standard error of the mean with p < 0.05 considered statistically significant.

### Data Availability

The datasets generated during and/or analyzed during the current study are available from the corresponding author on reasonable request.

## Electronic supplementary material

Supplementary Data

## References

[1] Nakayama K (1996). Differences in bone and vitamin D metabolism between primary hyperparathyroidism and malignancy-associated hypercalcemia. J. Clin. Endocrinol. Metab..

[2] Tangirala RK, Murao K, Quehenberger O (1997). Regulation of expression of the human monocyte chemotactic protein-1 receptor (hCCR2) by cytokines. J. Biol. Chem..

[3] Hopwood B, Tsykin A, Findlay DM, Fazzalari NL (2009). Gene expression profile of the bone microenvironment in human fragility fracture bone. Bone..

[4] Lu Y (2007). *C*CCR2 expression correlates with prostate cancer progression. J. Cell Biochem..

[5] Lu Y (2007). Monocyte chemotactic protein-1 mediates prostate cancer-induced bone resorption. Cancer Res..

[6] Lu Y (2007). PTHrP-induced MCP-1 production by human bone marrow endothelial cells and osteoblasts promotes osteoclast differentiation and prostate cancer cell proliferation and invasion *in vitro*. Int. J. Cancer..

[7] Kim MS, Day CJ, Morrison NA (2005). MCP-1 is induced by receptor activator of nuclear factor-{kappa}B ligand, promotes human osteoclast fusion, and rescues granulocyte macrophage colony-stimulating factor suppression of osteoclast formation. J. Biol. Chem..

[8] Kim, M. S. *et al*. MCP-1-induced human osteoclast-like cells are tartrate-resistant acid phosphatase, NFATc1, and calcitonin receptor-positive but require receptor activator of NFkappaB ligand for bone resorption. *J. Biol. Chem.* **281**, 1274–1285 (2006). 10.1074/jbc.M510156200 16280328

[9] Miyamoto, K. *et al*. MCP-1 expressed by osteoclasts stimulates osteoclastogenesis in an autocrine/paracrine manner. *Biochem. Biophys. Res. Commun.* **383**, 373–377 (2009). 10.1016/j.bbrc.2009.04.020 19364494

[10] Binder, N. B. *et al*. Estrogen-dependent and C-C chemokine receptor-2-dependent pathways determine osteoclast behavior in osteoporosis. *Nat. Med.* **15**, 417–424 (2009). 10.1038/nm.1945 19330010

[11] Li, X. *et al*. Determination of dual effects of parathyroid hormone on skeletal gene expression *in vivo* by microarray and network analysis. *J. Biol. Chem.* **282**, 33086–33097 (2007). 10.1074/jbc.M705194200 17690103

[12] Li, X. *et al*. Parathyroid hormone stimulates osteoblastic expression of MCP-1 to recruit and increase the fusion of pre/osteoclasts. *J. Biol. Chem.* **282**, 33098–33106 (2007). 10.1074/jbc.M611781200 17690108

[13] Tamasi, J. A. *et al*. Monocyte chemoattractant protein-1 is a mediator of the anabolic action of parathyroid hormone on bone. *J. Bone Miner. Res.* **28**, 1975–1986 (2013). 10.1002/jbmr.1933 PMC3749291 23519994

[14] Watson, P. H. *et al*. Enhanced osteoblast development after continuous infusion of hPTH(1-84) in the rat. *Bone* **24**, 89–94 (1999). 10.1016/s8756-3282(98)00170-7 9951775

[15] Lee SK, Lorenzo JA (1999). Parathyroid hormone stimulates TRANCE and inhibits osteoprotegerin messenger ribonucleic acid expression in murine bone marrow cultures: correlation with osteoclast-like cell formation. Endocrinology..

[16] Ma, Y. L. *et al*. Catabolic effects of continuous human PTH (1–38) *in vivo* is associated with sustained stimulation of RANKL and inhibition of osteoprotegerin and gene-associated bone formation. *Endocrinology.* **142**, 4047–4054 (2001). 10.1210/endo.142.9.8356 11517184

[17] Silverberg, S. J. *et al*. Skeletal disease in primary hyperparathyroidism. *J. Bone Miner. Res.* **4**, 283–291 (1989). 10.1002/jbmr.5650040302 2763869

[18] Walker, M. D. *et al*. Effect of renal function on skeletal health in primary hyperparathyroidism. *J. Clin. Endocrinol. Metab* **97**, 1501–1507 (2012). 10.1210/jc.2011-3072 PMC3339888 22399521

[19] Tsou, C. L. *et al*. Critical roles for CCR2 and MCP-3 in monocyte mobilization from bone marrow and recruitment to inflammatory sites. *J. Clin. Invest.* **117**, 902–909 (2007). 10.1172/JCI29919 PMC1810572 17364026

[20] Wishart J, Horowitz M, Need A, Nordin BE (1990). Relationship between forearm and vertebral mineral density in postmenopausal women with primary hyperparathyroidism. Arch. Intern. Med..

[21] Iida-Klein, A. *et al*. Short-term continuous infusion of human parathyroid hormone 1–34 fragment is catabolic with decreased trabecular connectivity density accompanied by hypercalcemia in C57BL/J6 mice. *J. Endocrinol.* **186**, 549–557 (2005). 10.1677/joe.1.06270 16135674

[22] Lotinun, S. *et al*. Continuous parathyroid hormone induces cortical porosity in the rat: effects on bone turnover and mechanical properties. *J. Bone Miner. Res.* **19**, 1165–1171 (2004). 10.1359/JBMR.040404 15177000

[23] Parisien, M. *et al*. The histomorphometry of bone in primary hyperparathyroidism: preservation of cancellous bone structure. *J. Clin. Endocrinol. Metab.* **70**, 930–938 (1990). 10.1210/jcem-70-4-930 2318948

[24] Zhou H, Shen V, Dempster DW, Lindsay R (2001). Continuous parathyroid hormone and estrogen administration increases vertebral cancellous bone volume and cortical width in the estrogen-deficient rat. J. Bone Miner. Res..

[25] Mosekilde L (2008). Primary hyperparathyroidism and the skeleton. Clin. Endocrinol. (Oxf)..

[26] Tournis S (2014). Effect of parathyroidectomy versus risedronate on volumetric bone mineral density and bone geometry at the tibia in postmenopausal women with primary hyperparathyroidism. J Bone Miner Metab..

[27] Kyriakides TR (2004). The CC chemokine ligand, CCL2/MCP1, participates in macrophage fusion and foreign body giant cell formation. Am. J. Pathol..

[28] Chang, M. K. *et al*. Osteal tissue macrophages are intercalated throughout human and mouse bone lining tissues and regulate osteoblast function *in vitro* and *in vivo*. *J. Immunol.* **181**, 1232–1244 (2008). 10.4049/jimmunol.181.2.1232 18606677

[29] Winkler, I. G. *et al*. Bone marrow macrophages maintain hematopoietic stem cell (HSC) niches and their depletion mobilizes HSCs. *Blood.* **116**, 4815–4828 (2010). 10.1182/blood-2009-11-253534 20713966

[30] Ashley, J. W. *et al*. Genetic ablation of CD68 results in mice with increased bone and dysfunctional osteoclasts. *PLoS. One.* **6**, e25838 (2011). 10.1371/journal.pone.0025838 PMC3185056 21991369

[31] Sul, O. J. *et al*. Absence of MCP-1 leads to elevated bone mass via impaired actin ring formation. *J. Cell Physiol.* **227**, 1619–1627 (2012). 10.1002/jcp.22879 21678414

[32] Li, C. Y. *et al*. Mice lacking cathepsin K maintain bone remodeling but develop bone fragility despite high bone mass. *J. Bone Miner. Res.* **21**, 865–875 (2006). 10.1359/jbmr.060313 16753017

[33] Pennypacker, B. *et al*. Bone density, strength, and formation in adult cathepsin K (^−/−^) mice. *Bone.* **44**, 199–207 (2009). 10.1016/j.bone.2008.08.130 18845279

[34] Walker, E. C. *et al*. Sustained RANKL response to parathyroid hormone in oncostatin M receptor-deficient osteoblasts converts anabolic treatment to a catabolic effect *in vivo*. *J. Bone Miner. Res.* **27**, 902–912 (2012). 10.1002/jbmr.1506 22190112

[35] Gu L (2000). Control of TH_2_ polarization by the chemokine monocyte chemoattractant protein-1. Nature.

[36] Lu B (1998). Abnormalities in monocyte recruitment and cytokine expression in monocyte chemoattractant 1-deficient mice. J. Exp. Med..

[37] Weisberg SP (2006). CCR2 modulates inflammatory and metabolic effects of high-fat feeding. J. Clin. Invest..

[38] Khan UA, Hashimi SM, Bakr MM, Forwood MR, Morrison NA (2016). CCL2 and CCR2 are essential for the formation of osteoclasts and foreign body giant cells. J. Cell Biochem..

[39] Morrison NA, Day CJ, Nicholson GC (2014). Dominant negative MCP-1 blocks human osteoclast differentiation. J. Cell Biochem..

[40] Kirk, P. S. *et al*. Inhibition of CCL2 signaling in combination with docetaxel treatment has profound inhibitory effects on prostate cancer growth in bone. *Int. J. Mol. Sci.* **14**, 10483–10496 (2013). 10.3390/ijms140510483 PMC3676850 23698775

[41] Zhang J, Lu Y, Pienta KJ (2010). Multiple roles of chemokine (C-C motif) ligand 2 in promoting prostate cancer growth. J. Natl. Cancer Inst..

[42] Zhang J, Patel L, Pienta KJ (2010). CC chemokine ligand 2 (CCL2) promotes prostate cancer tumorigenesis and metastasis. Cytokine Growth Factor Rev..

[43] Graves DT, Alsulaimani F, Ding Y, Marks SC (2002). Developmentally regulated monocyte recruitment and bone resorption are modulated by functional deletion of the monocytic chemoattractant protein-1 gene. Bone..

[44] Wise GE, Huang H, Que BG (1999). Gene expression of potential tooth eruption molecules in the dental follicle of the mouse. Eur. J. Oral Sci..

[45] Gao, Y. *et al*. T cells potentiate PTH-induced cortical bone loss through CD40L signaling. *Cell Metab.* **8**, 132–145 (2008). 10.1016/j.cmet.2008.07.001 PMC2569843 18680714

[46] Sukumar D, Partridge NC, Wang X, Shapses SA (2011). The high serum monocyte chemoattractant protein-1 in obesity is influenced by high parathyroid hormone and not adiposity. J. Clin. Endocrinol. Metab.

[47] Patel H, Trooskin S, Shapses S, Sun W, Wang X (2014). Serum monocyte chemokine protein-1 levels before and after parathyroidectomy in patients with primary hyperparathyroidism. Endocr. Pract..

[48] Glatt V, Canalis E, Stadmeyer L, Bouxsein ML (2007). Age-related changes in trabecular architecture differ in female and male C57BL/6J mice. J. Bone Miner. Res..

[49] Bouxsein, M. L. *et al*. Guidelines for assessment of bone microstructure in rodents using micro-computed tomography. *J. Bone Miner. Res.* **25**, 1468–1486 (2010). 10.1002/jbmr.141 20533309

[50] Siddiqui, J. A. *et al*. A naturally occurring rare analog of quercetin promotes peak bone mass achievement and exerts anabolic effect on osteoporotic bone. *Osteoporos. Int.* **22**, 3013–3027 (2011). 10.1007/s00198-010-1519-4 21225417

[51] Dempster, D. W. *et al*. Standardized nomenclature, symbols, and units for bone histomorphometry: a 2012 update of the report of the ASBMR Histomorphometry Nomenclature Committee. *J. Bone Miner. Res.* **28**, 2–17 (2013). 10.1002/jbmr.1805 PMC3672237 23197339

[52] Siddiqui JA (2010). 8,8″-Biapigeninyl stimulates osteoblast functions and inhibits osteoclast and adipocyte functions: Osteoprotective action of 8,8″-biapigeninyl in ovariectomized mice. Mol. Cell Endocrinol..

[53] Pfaffl MW (2001). A new mathematical model for relative quantification in real-time RT-PCR. Nucleic Acids Res..

