# Supplementary material for: Catabolic Effects of Human PTH (1–34) on Bone: Requirement of Monocyte Chemoattractant Protein-1 in Murine Model of Hyperparathyroidism
Source: Sci Rep. 2017 Nov 10;7:15300. doi: 10.1038/s41598-017-15563-7 (PMC5681546; doi:10.1038/s41598-017-15563-7)
Supplement: Supplementary file 1 — Supplementary Data [file 41598_2017_15563_MOESM1_ESM.pdf]

Catabolic Effects of Human PTH (1–34) on Bone: Requirement of Monocyte Chemoattractant Protein-1 in Murine Model of Hyperparathyroidism

Jawed A Siddiqui, Ph.D.[1], Joshua Johnson, M.S.[1], Carole Le Henaff, Ph.D.[1], Claudine L. Bitel, Ph.D.[1],

Joseph A Tamasi, Ph.D.[2] and Nicola C. Partridge, Ph.D.[1]

Supplementary Figure 1

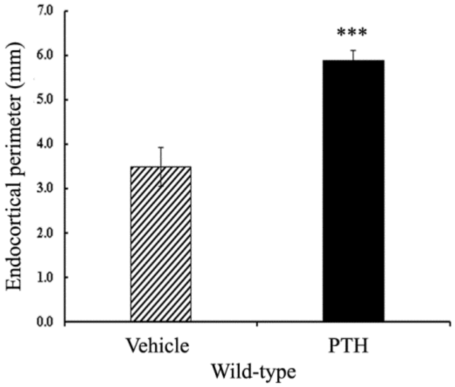

Supplementary Figure 1. Endocortical Perimeter of WT Mice Receiving cPTH for 14 days. PTH-treated mice show a significant increase in endocortical perimeter, ***p<0.001, compared with the vehicle-treated animals, indicating cortical resorption is taking place.

Supplementary Figure 2

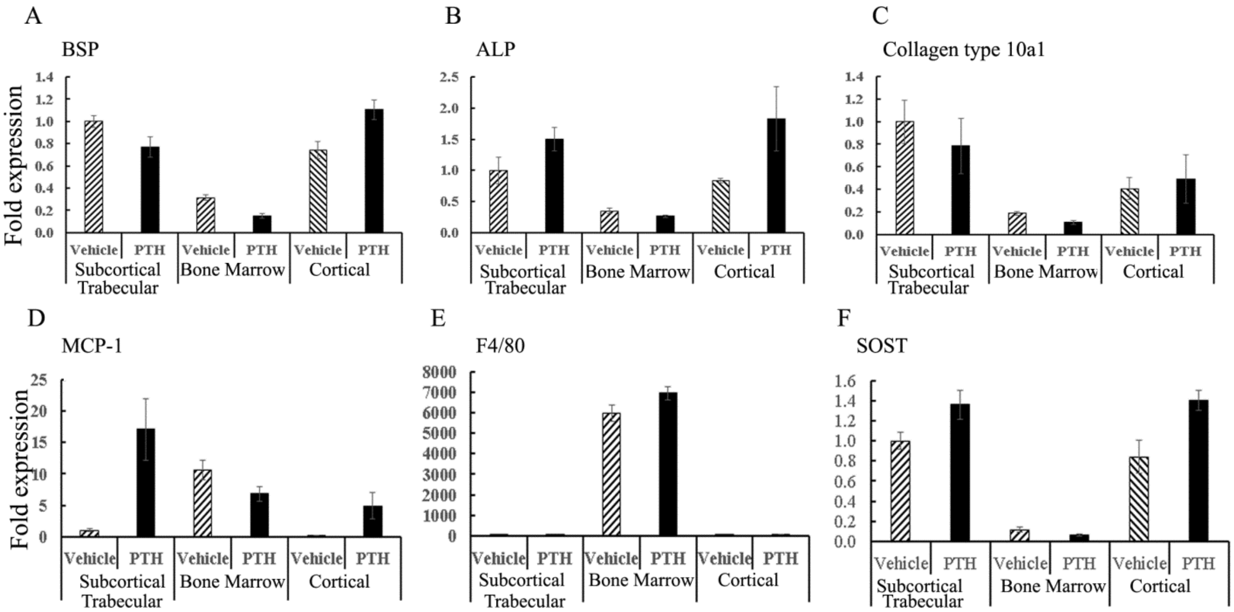

Supplementary Figure 2. mRNA Expression in the Subcortical Trabecular Bone, Bone Marrow and Cortical Bone of WT Mice Receiving cPTH for 14 Days. **A**, BSP, bone sialoprotein; **B**, ALP, alkaline phosphatase; **C**, Collagen type 10a1; **D**, MCP-1, monocyte chemoattractant protein-1; **E**, F4/80; **F**, SOST, sclerostin gene.
